# Post-vaccination infection rates and modification of COVID-19 symptoms in vaccinated UK school-aged children and adolescents: A prospective longitudinal cohort study

**DOI:** 10.1016/j.lanepe.2022.100429

**Published:** 2022-07-08

**Authors:** Erika Molteni, Liane S. Canas, Kerstin Kläser, Jie Deng, Sunil S. Bhopal, Robert C. Hughes, Liyuan Chen, Benjamin Murray, Eric Kerfoot, Michela Antonelli, Carole H. Sudre, Joan Capdevila Pujol, Lorenzo Polidori, Anna May, Prof Alexander Hammers, Jonathan Wolf, Prof Tim D. Spector, Claire J. Steves, Prof Sebastien Ourselin, Michael Absoud, Marc Modat, Prof Emma L. Duncan

**Affiliations:** a School of Biomedical Engineering & Imaging Sciences, King's College London, London, UK; b Population Health Sciences Institute, Faculty of Medical Sciences, Newcastle University, Newcastle upon Tyne, Tyne and Wear, UK; c Department of Population Health, Faculty of Epidemiology & Population Health, London School of Hygiene & Tropical Medicine, Keppel Street, London, UK; d Zoe Limited, London, UK; e Department of Twin Research and Genetic Epidemiology, King's College London, London, UK; f Department of Aging and Health, Guy's and St Thomas’ NHS Foundation Trust, London, UK; g Children's Neurosciences, Evelina London Children's Hospital, St Thomas’ Hospital, King's Health Partners, Academic Health Science Centre, London, UK; h Department of Women and Children's Health, Faculty of Life Sciences and Medicine, School of Life Course Sciences, King's College London, London, UK; i Department of Endocrinology, Guy's and St Thomas’ NHS Foundation trust, London, UK; j MRC Unit for Lifelong Health and Ageing, Department of Population Health Sciences and Centre for Medical Image Computing, Department of Computer Science, University College London, London, UK

**Keywords:** SARS-CoV-2 vaccination, COVID-19 vaccination, BNT162b2 vaccine effectiveness, SARS-CoV-2 vaccination in children, Paediatrics, BNT162b2, Comirnaty SARS-CoV-2 vaccine (BioNTech, Pfizer), CA, Children and adolescents, KCL, King's College London, LFAT, Lateral flow antigen test, OR, Odds Ratio, PCR, Polymerase chain reaction, SARS-CoV-2, Severe acute respiratory syndrome‐related coronavirus 2, UK, United Kingdom of Great Britain and Northern Ireland

## Abstract

**Background:**

We aimed to explore the effectiveness of one-dose BNT162b2 vaccination upon SARS-CoV-2 infection, its effect on COVID-19 presentation, and post-vaccination symptoms in children and adolescents (CA) in the UK during periods of Delta and Omicron variant predominance.

**Methods:**

In this prospective longitudinal cohort study, we analysed data from 115,775 CA aged 12-17 years, proxy-reported through the Covid Symptom Study (CSS) smartphone application. We calculated post-vaccination infection risk after one dose of BNT162b2, and described the illness profile of CA with post-vaccination SARS-CoV-2 infection, compared to unvaccinated CA, and post-vaccination side-effects.

**Findings:**

Between August 5, 2021 and February 14, 2022, 25,971 UK CA aged 12-17 years received one dose of BNT162b2 vaccine. The probability of testing positive for infection diverged soon after vaccination, and was lower in CA with prior SARS-CoV-2 infection. Vaccination reduced proxy-reported infection risk (-80·4% (95% CI -0·82 -0·78) and -53·7% (95% CI -0·62 -0·43) at 14–30 days with Delta and Omicron variants respectively, and -61·5% (95% CI -0·74 -0·44) and -63·7% (95% CI -0·68 -0.59) after 61–90 days). Vaccinated CA who contracted SARS-CoV-2 during the Delta period had milder disease than unvaccinated CA; during the Omicron period this was only evident in children aged 12-15 years. Overall disease profile was similar in both vaccinated and unvaccinated CA. Post-vaccination local side-effects were common, systemic side-effects were uncommon, and both resolved within few days (3 days in most cases).

**Interpretation:**

One dose of BNT162b2 vaccine reduced risk of SARS-CoV-2 infection for at least 90 days in CA aged 12-17 years. Vaccine protection varied for SARS-CoV-2 variant type (lower for Omicron than Delta variant), and was enhanced by pre-vaccination SARS-CoV-2 infection. Severity of COVID-19 presentation after vaccination was generally milder, although unvaccinated CA also had generally mild disease. Overall, vaccination was well-tolerated.

**Funding:**

UK Government Department of Health and Social Care, Chronic Disease Research Foundation, The Wellcome Trust, UK Engineering and Physical Sciences Research Council, UK Research and Innovation London Medical Imaging & Artificial Intelligence Centre for Value Based Healthcare, UK National Institute for Health Research, UK Medical Research Council, British Heart Foundation and Alzheimer's Society, and ZOE Limited.

**Research in context**

**Evidence before this study**

We searched PubMed database for peer-reviewed articles and medRxiv for preprint papers, published between January 1, 2021 and February 15, 2022 using keywords (“SARS-CoV-2” OR “COVID-19”) AND (child* OR pediatric* OR teenager*) AND (“vaccin*” OR “immunization campaign”) AND (“efficacy” OR “effectiveness” OR “symptoms”) AND (“delta” or “omicron” OR “B.1.617.2” OR “B.1.1.529”). The PubMed search retrieved 36 studies, of which fewer than 30% specifically investigated individuals <18 years, and ∼70% were prospective controlled or clinical trials, or national surveys, overall producing high evidence.

Eleven studies explored SARS-CoV-2 viral transmission: seroprevalence in children (*n* = 4), including age-dependency of susceptibility to SARS-CoV-2 infection (*n* = 1), SARS-CoV-2 transmission in schools (*n* = 5), and the effect of school closure on viral transmission (*n* = 1).

Eighteen documents reported clinical aspects, including manifestation of infection (*n* = 13), symptomatology, disease duration, and severity in children. Other studies estimated emergency department visits, hospitalization, need for intensive care, and/or deaths in children (*n* = 4), and explored prognostic factors (*n* = 1).

Thirteen studies explored vaccination-related aspects, including vaccination of children within specific paediatric co-morbidity groups (e.g., children with Down syndrome, inflammatory bowel disease, and cancer survivors, *n* = 4), mRNA vaccine effectiveness or efficacy in children and adolescents from the general population (*n* = 7), and the relation between vaccination and severity of disease and hospitalization cases (*n* = 2).

Four clinical trials were conducted using mRNA vaccines in minors, also exploring side effects. Sixty percent of children were found to have side effects after BNT162b2 vaccination, and especially after the second dose; however, most symptoms were mild and transient apart from rare uncomplicated skin ulcers. Two studies focused on severe adverse effects and safety of SARS-CoV-2 vaccines in children, reporting on myocarditis episodes and two cases of Guillain-Barrè syndrome. All other studies were beyond the scope of our research.

**Added value of this study**

We assessed multiple components of the effects of the UK vaccination campaign in a cohort of children and adolescents (CA) aged 12-17 years drawn from a large UK community-based citizen-science study, who had received a first dose of BNT162b2 vaccine. We describe a variant-dependent protective effect of the first dose against both Delta and Omicron, with additional protective effect of pre-vaccination SARS-CoV-2 infection on post-vaccination re-infection. We compare the illness profile in CA infected post-vaccination with that of unvaccinated CA, showing overall milder disease with fewer symptoms in vaccinated CA. We describe local and systemic side-effects during the first week following first-dose vaccination, illustrating that local symptoms are common, systemic symptoms uncommon, and both usually transient.

**Implications of all the available evidence**

Our data show that the first dose BNT162b2 vaccination in CA is associated with reduction in risk of infection by SARS-CoV-2 variants, with generally local and brief side-effects. If infected after vaccination, COVID-19 is milder, if manifest at all. The study aims to contribute quantitative evidence to the risk-benefit evaluation of vaccination in CA to inform discussion regarding rationale for their vaccination and the designing of national immunisation campaigns for this age group; and applies citizen-science approaches in the conduct of epidemiological surveillance and data collection in the UK community.

Importantly, this study was conducted during Delta and Omicron predominance in UK; specificity of vaccination effectiveness to variants is also illustrated; and results may not be generalizable to future SARS-CoV-2 strains.

Alt-text: Unlabelled box

## Introduction

SARS-CoV-2 infection in children and adolescents (CA) is asymptomatic in approximately 50% of cases^1^^,^^2^; and in symptomatic cases is usually mild and of short duration, with low risk of hospitalisation or death.^3^^,^^4^ In the general population, mass vaccination against SARS-CoV-2 is a key strategy to reduce morbidity and mortality of COVID-19, and manage demand on healthcare systems.^5^ Considering vaccination in children specifically, two-dose vaccination with BNT162b2 was highly efficacious in reducing infection risk in children aged 5-15 years, with most side-effects mild and short-lived, in studies conducted pre-Delta variant predominance.^6^^,^^7^ However, concerns regarding vaccine-related risk of myo- and peri-carditis, particularly in boys and young men, have subsequently emerged.^8^, ^9^, ^10^

SARS-CoV-2 vaccination campaigns in CA have varied internationally. Evaluating vaccination for children includes consideration of the effect of vaccination upon susceptibility and severity of SARS-CoV-2 infection, the strong correlation of age and COVID-19 severity, and the educational and psychosocial effects of ongoing outbreaks, lock-downs, and quarantine policies (e.g., https://www.cdc.gov/coronavirus/2019-ncov/vaccines/recommendations/children-teens.html). In the UK, universal single-dose vaccination was authorized for the BNT162b2 vaccine for adolescents aged 16-17 years on August 4, 2021 (https://www.gov.uk/government/news/jcvi-issues-updated-advice-on-covid-19-vaccination-of-young-people-aged-16-to-17) and extended to children aged 12-15 years on September 13, 2021 (https://www.gov.uk/government/publications/universal-vaccination-of-children-and-young-people-aged-12-to-15-years-against-covid-19/universal-vaccination-of-children-and-young-people-aged-12-to-15-years-against-covid-19) with primary care advice received shortly thereafter. When these policies were announced, Delta (B.1.617.2) was the predominant circulating variant in the UK. After November 27, 2021, the Omicron (B.1.1.529) variant emerged and spread rapidly, becoming the predominant variant in the UK after December 20, 2021 (https://assets.publishing.service.gov.uk/government/uploads/system/uploads/attachment_data/file/1043807/technical-briefing-33.pdf).

Estimates of vaccination effectiveness in real-world settings may differ from trial data, with changes over time in exposure, prior infection-induced immunity, emergence of new variants, testing policies, and ‘track and trace’ strategies. Real-world studies in individuals aged 12-18 years confirmed high BNT162b2 vaccination effectiveness against severe COVID-19 presentation.^11^ However, data on vaccination effect upon community-managed infections, and on symptom presentation, are less clear for the school-aged population. Moreover, effectiveness of vaccination against newer SARS-CoV-2 variants remains incompletely determined.^12^

Citizen science allows capturing of phase 4 data for medicinal products after their pivotal phase 3 trials, allowing real-time and real-world monitoring of effectiveness and side-effect profile, at scale.^13^^,^^14^ Here, using such an approach, we aimed to explore effectiveness of first-dose BNT162b2 vaccination (i.e., the UK vaccination policy in CA at the time of this study) upon risk of subsequent SARS-CoV-2 infection for individuals aged 12 to 17 years. We considered also the effects for two variants, Delta and Omicron, and of SARS-CoV-2 infection prior to first-dose vaccination. We also studied the effect of vaccination on COVID-19 presentation, including hospital presentation; and post-vaccination symptomatology.

## Methods

This prospective observational study used data from the COVID Symptom Study, collected through a smart-phone application: the ZOE COVID Study App (details previously published^14^). Adult participants can regularly self-report COVID-19 related information through the smartphone application. Adult participants can also proxy-report for others, including children and adolescents (typically sons and daughters living in the same household although data cannot be directly linked between the adult contributor and the proxy-reported individual). Adults can proxy-report symptoms any time, including responses to direct questions (Supplementary Table 1) and free-text, any SARS-CoV-2 testing and results, vaccination, post-vaccination symptoms (Supplementary Table 2), and health care access, as well as demographic and co-morbidity data (the latter mainly informed by common adult co-morbidities). Daily reporting is encouraged by the application. Teenagers aged 16-17 can also self-report.

Ethics approval was granted by the KCL Ethics Committee (LRS-19/20-18210). Upon registration with the app, all participants were provided with study information, and gave consent for their data to be used for COVID-19 research. Governance was specifically granted to enable use of proxy-reported data, which included data from children. Research was conducted in full compliance with the Declaration of Helsinki and further updates.

Data from all UK individuals aged 12-17 years proxy-reported by an adult were considered. All self-reported data were excluded. Individuals aged 12 to 15 years were included in the *children* group; individuals aged 16 to 17 years constituted the *adolescents* group. Data were considered from August 5, 2021 to February 14, 2022. The start date was the day after release of the policy for universal vaccination of teenagers aged 16-17 years in UK. The end date corresponded to data analysis initiation (census date). Data were extracted and curated through ExeTera software.^15^ Exclusion of self-reported data avoided potential duplicate data entry (i.e., from self- and proxy-reporting of older teenagers) and uncertainty due to lower perseverance in self-reporting in this group, as described previously.^3^

All analyses described herein refer to single first dose BNT162b2 vaccination, if received, administered between August 5, 2021 and February 14, 2022. Some vulnerable CA and those living with vulnerable adults were eligible to access vaccination earlier (July 19, 2021) and/or receive two vaccine doses (https://www.gov.uk/government/news/jcvi-issues-advice-on-covid-19-vaccination-of-children-and-young-people); data from these individuals were excluded by constraint upon vaccination date (i.e., after policy announcement dates, as above) and/or by vaccination dose number (i.e., only single/first dose data were considered).

SARS-CoV-2 infection was defined as a proxy-logged positive result from either a reverse transcription polymerase chain reaction (PCR) test or lateral flow antigen test (LFAT). A declaration of previous infection at time of first registration in the app, with or without confirmatory test information, was also accepted. Testing for SARS-CoV-2 was per local guidelines, and may have been prompted by symptoms, school and/or other social requirements, contact tracing, or other reason; rationale for testing for proxy-reported individuals was not captured.

*Prior* SARS-CoV-2 infection was defined as a positive test for SARS-CoV-2 assessed as above, and proxy-logged before a critical event such as *prior* to date of vaccination, or to date of matching for unvaccinated controls as per each analysis.

When conducting analyses during the periods of Delta and Omicron variant prevalence in UK, data were subset within two further time frames: August 5, 2021 to November 27, 2021, when Delta was the predominant circulating SARS-CoV-2 variant (minimum prevalence >70%); and December 20, 2021 to February 14, 2022, when Omicron was prevailing (minimum prevalence >70%) (https://www.gisaid.org/hcov19-variants/).

**Figure 1:**
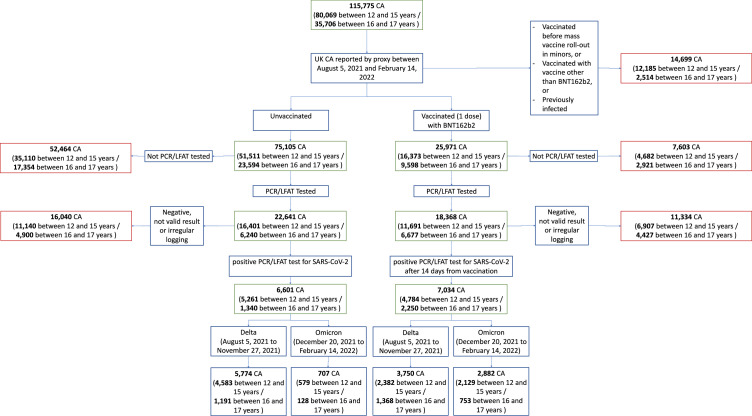
Flowchart of participants according to inclusion and exclusion criteria for this study. Entire cohort is given first, before subdivision into vaccinated and unvaccinated CA. Note here the two age groups had differing time periods for data consideration (adolescents aged 16–17 years: able to receive vaccination after August 4, 2021; children aged 12-15 years: able to receive vaccination after September 18, 2021). Further subdivision provides numbers of individuals for periods of Delta and Omicron variant predominance in UK. Considered time frames for Delta and Omicron variant predominance were not contiguous; thus, numbers of CA within each time frame do not sum to numbers across the entire study period. Not valid result = test result proxy-reported as “failed test” or “still waiting”. Irregular logging = proxy-reporting with intervals of more than 7 days between proxy-reports during illness. PCR – polymerase chain reaction. LFAT – lateral flow antigen test.

Study design is depicted in Figure 1. For each sub-analysis, criteria for the subject selection and resulting sample sizes are reported in Supplementary Table 3.

### Risk (hazard) of post-vaccination infection in vaccinated and unvaccinated CA without prior SARS-CoV-2 infection

To study the risk of post-vaccination infection, we assessed the effect of vaccination upon remaining negative when tested for infection (*survival*) in SARS-CoV-2-naïve individuals, during the periods of Delta and Omicron variant predominance in UK, as defined above and in Supplementary Table 3. We matched 1:1 vaccinated and unvaccinated CA for week of test, sex, and age, excluding CA with prior SARS-CoV-2 infection. For this analysis, *no prior infection* was defined in vaccinated CA as no infection before vaccination and for unvaccinated CA as no prior infection before week of matching. We constructed Kaplan-Meier survival curves for SARS-CoV-2 testing, with the first positive test being the failure event.

### Effect of pre-vaccination SARS-CoV-2 infection on post-vaccination infection risk

To assess the effect of prior SARS-CoV-2 infection on post-vaccination infection risk, we matched 1:1 vaccinated CA with vs. without prior SARS-CoV-2 infection (matched for week of test, sex, and age). Kaplan-Meier survival curves were constructed. Analysis considered periods of Delta and Omicron variant predominance, as defined above.

### Single dose BNT162b2 vaccination effectiveness

To assess the real-world effectiveness of one-dose BNT162b2 vaccination, infections in vaccinated and unvaccinated CA naïve to SARS-CoV-2 were compared during the periods of Delta and Omicron variant predominance in UK. This analysis is explained in detail in Supplementary Material 1. Sample sizes are reported in Supplementary Table 3.

### Illness profile of first SARS-CoV-2 infection in vaccinated and unvaccinated CA

We assessed symptom manifestation and illness burden in previously SARS-CoV-2-naïve CA, from when they first tested positive, including symptomatic and asymptomatic CA. We compared illness profiles, including symptoms and disease burden, of one-dose vaccinated and unvaccinated CA. Symptoms were considered from one week before to one month after a positive test, with the additional constraint that symptoms had to commence between one week before and two weeks after the positive test result, as previously defined.^3^ Disease burden was computed as the median count of individual symptoms per week. We calculated illness duration from the first day of first symptom to the last day of last symptom; and we assessed prevalence and distribution of duration for each symptom.^3^

To compare the illness profile in vaccinated vs. unvaccinated CA without prior infection, we matched individuals for week of test, sex and age.^3^ Matching was performed by Euclidean distance, with equal number of subjects in each population when the number of unvaccinated subjects was sufficient; however, this was not possible for all comparisons due to the rapidly declining numbers of unvaccinated CA over time. Asymptomatic subjects who tested positive were included in this analysis. As previously,^16^ after adjustment for sex, age, and number of comorbidities, odds ratios (ORs) were computed for each symptom between the two groups of vaccinated and unvaccinated CA, with significance adjusted for False Discovery Rate. Hospital presentation (emergency room presentation and/or hospital admission) was also assessed, noting here that we did not have health records linkage and thus could not determine reason for presentation.

### Post-vaccination symptoms

We assessed post-vaccination symptoms in all vaccinated CA. For this analysis only, we excluded CA with a positive SARS-CoV-2 test within three months prior to vaccination, so that reported symptoms pertained to vaccination *per se* rather than residual COVID-19. As previously,^17^ we counted the number of individuals experiencing any symptom in the seven days after vaccination, assessing daily prevalence of pre-specified systemic (Supplementary Table 1) and local (Supplementary Table 2) symptoms during this time period.

A technical issue in app software occurred on August 5, 2021 which prevented recording of local symptoms: this was resolved by August 23, 2021. Thus, CA due to report post-vaccination local symptoms within this time window (*n* = 357) were removed from the analysis of local symptoms; however, systemic symptoms were still able to be assessed throughout. Notwithstanding this technical issue, reporting of systemic and/or local symptoms could occur independently; thus, the numbers of CA assessed for systemic and local symptoms differ even outside the August 5-23 window, as reported in Table 3 and Supplementary Table 3.

Lastly, free text field entries were analysed for symptoms not queried through the standard questions. Severe side-effects of BNT162b2 vaccine reported in the literature were specifically assessed within the free text, including anaphylaxis, thrombosis, cytopenia, myocarditis, pericarditis, Guillain-Barré syndrome, and (multi)organ failure.^8^, ^9^, ^10^^,^^18^

### Role of the funding source

The study funders had no role in the study design, contributors’ enrolment, data analysis, interpretation, or report writing.

## Results

### Cohort Description

The overall cohort comprised 25,971 vaccinated and 75,105 unvaccinated CA: 9598 vaccinated and 23,594 unvaccinated adolescents (aged 16-17 years) proxy-reported between August 5, 2021 and February 14, 2022; and 16,373 vaccinated and 51,511 unvaccinated children (aged 12-15 years) between September 18, 2021 and February 14, 2022 (Figure 1, Table 1).

**Table 1:** Demographic characteristics of UK CA proxy-logged as part of the COVID Symptom Study between August 5, 2021 and February 14, 2022.

	Vaccinated CA (first dose BNT162b2)		Unvaccinated CA	
	*Children (aged 12-15 years)*	*Adolescents (aged 16-17 years)*	*Children (aged 12-15 years)*	*Adolescents (aged 16-17 years)*
Number of CA proxy-reported within relevant time frames*	16,373	9598	51,511	23,594
**Number of CA tested for SARS-CoV-2 (n)**	11,691	6677	16,401	6,240
#**Positive test result (n (%))**	4784 (40·9)	2250 (33·7)	5261 (32.1)	1340 (21·5)
#**Age (mean (SD))**	13.58 (1·09)	16.46 (0·50)	13·54 (1·11)	16·43 (0·50)
#**BMI (mean (SD))**	19·66 (3·67)	20.80 (3·86)	19·75 (3·76)	20·72 (3·82)
#**Male (n (%))**	6105 (52·2)	3508 (52·5)	8615 (52·5)	3310 (53·0)
#**CA with at least one comorbidity (n (%))**	644 (5·5)	362 (5·4)	917 (5·6)	340 (5·4)

⁎ Time frames: For adolescents aged 16-17 years, data were considered between August 5, 2021 and February 14, 2022. For children aged 12-15 years, data were considered between September 18, 2021 and February 14, 2022.

# These figures were calculated for those CA with least one SARS-CoV-2 test. BMI, body mass index. SD, standard deviation.

Data from 6677 vaccinated adolescents with at least one post-vaccination PCR or LFAT test were compared to 6240 unvaccinated adolescents analogously proxy-reported between August 5, 2021 and February 14, 2022. Similarly, data from 11,691 vaccinated children with at least one test post-vaccination were compared to 16,401 unvaccinated children, between September 18, 2021 and February 14, 2022. Significantly fewer unvaccinated CA were tested, compared with vaccinated CA (Table 1: assessed by Chi-squared testing; p<0.00001 for both adolescents and children). Other demographics are presented in Table 1. Cohort descriptions and sample size for each analysis are detailed in Supplementary Table 3.

### Risk (hazard) of post-vaccination infection in vaccinated and unvaccinated CA without prior SARS-CoV-2 infection

**Figure 2:**
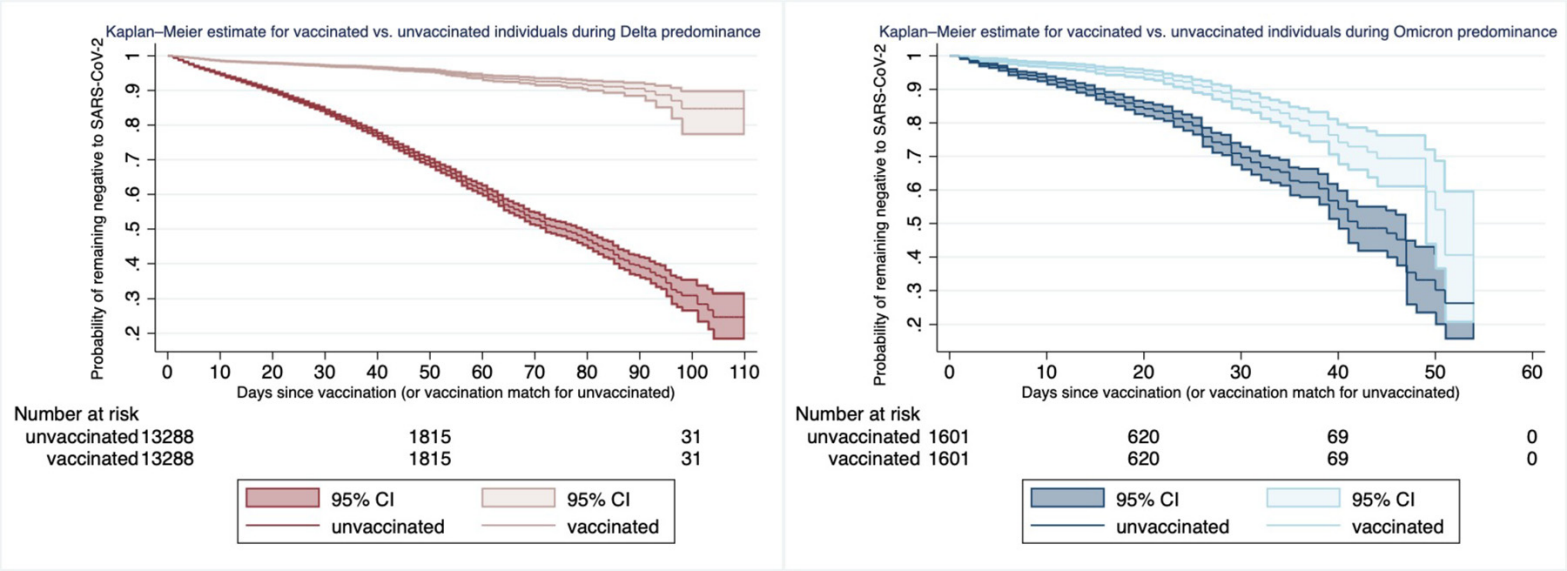
**Survival analysis**: Kaplan-Meier plots showing probability of reporting no positive result when tested for SARS-CoV-2 infection over time, for vaccinated and unvaccinated CA without prior SARS-CoV-2 infection, during periods of Delta (left panel) and Omicron (right panel) variant predominance.

A protective effect of vaccination on SARS-CoV-2 infection was evident, with risk quickly diverging between vaccinated and unvaccinated CA as shown in the Kaplan-Meier survival analysis (Figure 2).

In CA without prior SARS-CoV-2 infection during Delta predominance, the probability of not reporting any positive SARS-CoV-2 infection result (i.e., assumption of remaining infection-free) post-vaccination was high: >90% for 11 weeks post-vaccination, decreasing to just below 90% at 90 days (Figure 2: left panel). During Omicron predominance, the probability of not reporting any positive SARS-CoV-2 infection result post-vaccination was shorter: >90% until 25 days post-vaccination, <90% at 30 days, and <80% at 40 days. Additionally, the vaccine-induced risk reduction was smaller for vaccinated vs. unvaccinated CA during Omicron vs. Delta periods.

### Effect of pre-vaccination SARS-CoV-2 infection on post-vaccination infection risk

**Figure 3:**
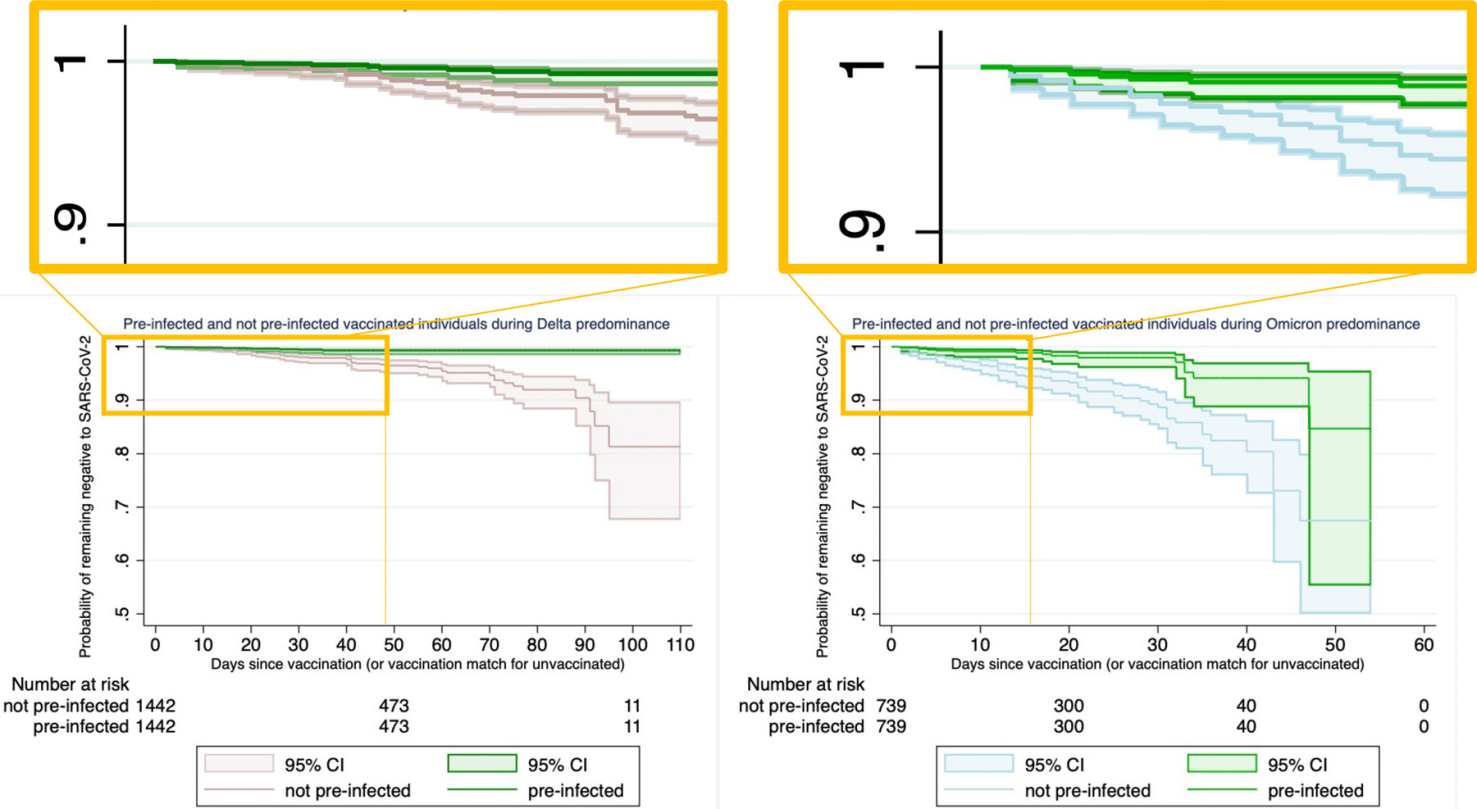
**Survival analysis:** Kaplan-Meier plots showing the probability of reporting no positive result at tests for SARS-CoV-2 infection over time for vaccinated CA with or without SARS-CoV-2 infection prior to vaccination, during periods of Delta (left panel) and Omicron (right panel) variant predominance.

Considering vaccinated CA with vs. without prior SARS-CoV-2 infection: prior infection enhanced the protective effect of vaccination against infection. During Delta predominance, the risk of reinfection was close to null beyond 100 days (Figure 3, left panel). During Omicron predominance, prior infection continued to confer enhanced vaccination effectiveness; however, data were insufficient to provide a robust quantitative description (Figure 3, right panel).

### Single dose BNT162b2 vaccination effectiveness

The adjusted risk ratio of infection post-vaccination vs no vaccination for Delta and Omicron are presented in Supplementary Figure 1. Breakdowns by age groups for Delta and Omicron periods are shown in Supplementary Figures 2 and 3 respectively. Comparing periods of Delta and Omicron predominance, risk reduction after vaccination appeared higher during Delta compared to Omicron, at least for the first three months in adolescents aged 16-17 years (Supplementary Figure 2) and at least for the first two months in children aged 12-15 years (Supplementary Figure 3).

### Illness profile of first SARS-CoV-2 infection in vaccinated and unvaccinated CA

**Figure 4:**
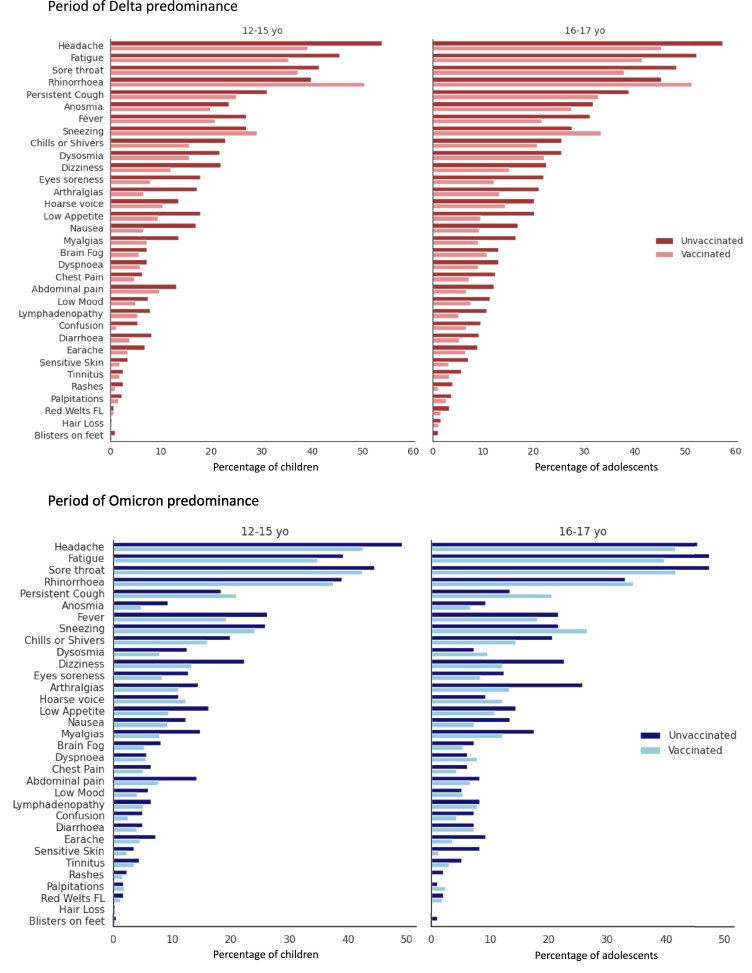
Illness profile in vaccinated and unvaccinated CA testing positive for SARS-CoV-2 during periods of Delta (top panels) and Omicron (bottom panels) variant predominance, in previously SARS-CoV-2-naÿve children (left panels) and adolescents (right panels). Asymptomatic CA are included in this figure.

**Figure 5:**
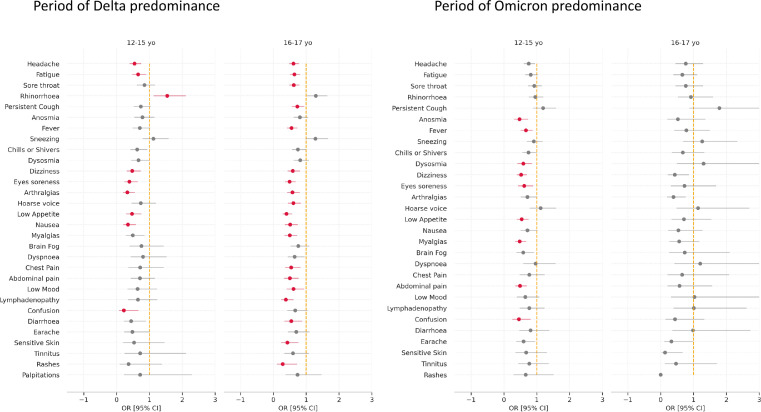
Odds ratios for symptom prevalence in vaccinated vs. unvaccinated CA first testing positive for SARS-CoV-2 during periods of Delta (left) and Omicron (right) variant predominance. Vaccinated CA had no reported SARS-CoV-2 infection prior to vaccination. Asymptomatic CA testing positive for SARS-CoV-2 are included in the computation. Red colour encodes significant p-values after false discovery rate correction (alpha =0.05).

During Delta predominance, all symptoms except rhinorrhoea and sneezing had lower prevalence in CA infected post-vaccination (*n* = 3750) compared to infected unvaccinated CA (*n* = 5774) (Figure 4, top panel), matched for age, gender, and BMI. Statistical testing showed lower odds ratios for multiple symptoms in vaccinated CA including headache, fatigue, dizziness, eye soreness, arthralgias, low appetite, nausea; additionally, vaccinated children had lower odds for confusion and vaccinated adolescents had lower odds for multiple other symptoms including sore throat, persistent cough, fever, hoarse voice, myalgias, chest pain, abdominal pain, low mood, lymphadenopathy, diarrhoea, and rashes (Figure 5, left panel; Supplementary Table 4; alpha=0·05).

Similarly, during Omicron predominance, most symptoms had lower prevalence in vaccinated (*n* = 2882) compared to unvaccinated (*n* = 707) CA, except persistent cough and hoarse voice, and, in adolescents only, rhinorrhoea, sneezing, dysosmia, dyspnoea, and low mood, noting the marginal difference in these latter infrequent symptoms (Figure 4, bottom panel). Statistical testing showed lower odds ratios for anosmia, fever, dysosmia, dizziness, eye soreness, low appetite, myalgias, abdominal pain and confusion in children, but no significant differences for any symptom in adolescents (Figure 5, right panel; Supplementary Table 4).

For both Delta and Omicron periods, median symptom duration was 1 day for most symptoms, in both vaccinated and unvaccinated CA, other than very rare cutaneous manifestations (welts, blisters, hair loss), and eye soreness in adolescents with Omicron infection (Supplementary Figure 4). Overall, symptom persistence was similar during periods of Delta and Omicron predominance and for vaccinated and unvaccinated CA (Supplementary Figures 5 and 6 for children and adolescents, respectively).

Burden of illness was assessed for the first week and for the first 28 days. This analysis incorporated both symptomatic and asymptomatic test-positive individuals (Table 2: descriptive data only). During Delta predominance, vaccinated CA had slightly fewer symptoms than unvaccinated CA both for the first week (4 vs. 6 in children; 5 vs. 7 in adolescents) and for the first 28 days (3 vs. 4·75 in children; 4·25 vs. 5 in adolescents); total illness duration was similar. During Omicron predominance, symptom burden appeared globally slightly less than during the Delta period (Table 2), and illness duration was comparable. Symptom burden and illness duration were similar for vaccinated and unvaccinated CA.

**Table 2:** Illness burden and duration (including hospitalisation numbers) for vaccinated and unvaccinated CA with symptomatic first SARS-CoV-2 infection, for periods of Delta and Omicron variant predominance, and for the entire study period. Vaccinated CA were considered from at least 14 days post-vaccination. Median and [IQR] are reported for illness burden (symptom count) and duration (days). Numbers (percentage) are reported for hospital presentation; CA with a positive SARS-CoV-2 test may have presented to hospital for reasons other than COVID-19. Time frames for Delta and Omicron periods were not contiguous; thus, the joint two time periods do not correspond to the entire time period.

		Delta Period (August 5 – November 27, 2021)				Omicron Period (December 20, 2021 – February 14, 2022)				Entire time period of study (August 5, 2021 – February 14, 2022)			
		Symptoms during first 7 days of illness (count, [IQR])	Symptoms during first 28 days of illness (count [IQR])	Duration of illness ((median [IQR])	CA presenting to hospital* (n (%))	Symptoms during first 7 days of illness (count, [IQR])	Symptoms during First 28 days of illness (count [IQR])	Duration of illness (median [IQR])	CA presenting to hospital* (n (%))	Symptoms during first 7 days of illness (count, [IQR])	Symptoms during first 28 days of illness (count [IQR])	Duration of illness (median [IQR])	CA presenting to hospital* (n (%))
**Children: 12-15 years**	**Vaccinated**	4 symptoms [2; 7]	3 symptoms [2; 4]	2 days [1; 5]	1 child (0·31%)	4 symptoms [1; 7]	2 symptoms [2, 3]	2 days [1; 5]	9 children (0·71%)	4 symptoms [1; 7]	2.5 symptoms [2; 3·75]	2 days [1; 5]	13 children (0·64%)
	**Unvaccinated**	6 symptoms [2·25; 10]	4·75 symptoms [3·25; 5·38]	1 day [1; 5]	9 children (2·81%)	4 symptoms [1; 7]	3 symptoms [2; 4]	1 day [1; 4]	4 children (0·98%)	5 symptoms [2; 8]	4 symptoms [3·25; 5]	2 days [1; 6]	32 children (1·58%)
**Adolescents: 16-17 years**	**Vaccinated**	5 symptoms [2; 8]	4.25 symptoms [3; 5]	3 days [1; 7]	15 adolescents (2·66%)	4 symptoms [0; 7]	3 symptoms [1; 4]	1 day [1; 4]	1 adolescent (0·60%)	4 s symptoms [1·5; 8]	3.5 symptoms [3; 4]	2 days [1; 7]	17 adolescents (2·13%)
	**Unvaccinated**	7 symptoms [4; 11]	5 symptoms [4·25; 6·75]	3 days [1; 8]	9 adolescents (1·60%)	5 symptoms [2·5; 10]	3 symptoms [0; 5]	1 day [1; 3]	1 adolescent (1·03%)	7 symptoms [3; 11]	5 symptoms [4·25; 6·38]	3 days [1; 7]	12 adolescents (1·50%)

Very few CA were reported as having presented to the hospital during the entire study period. During the Delta period, 16 (1·8%) of 884 vaccinated CA were reported as having presented to hospital, compared to 18 (2·1%) of 864 unvaccinated CA. During the Omicron period, 10 (0·7%) of 1435 vaccinated CA were reported having presented to hospital, compared to 5 (1·0%) of 506 unvaccinated CA (Table 2). No statistical tests are possible for such low frequency data; and we are unable to determine reason for presentation (i.e., SARS-CoV-2 diagnosis may have been incidental).

### Post-vaccination symptoms

The prevalence of adverse events suggestibly attributable to vaccination is shown in Table 3.

**Table 3:** Symptoms reported at least once during the 7 days after vaccination, in vaccinated CA. Subjects positive to SARS-CoV-2 test prior to vaccination were excluded.

GENERAL POPULATION CHARACTERISTICS		
	Children (aged 12-15 years)	Adolescents (aged 16-17 years)
	**SYSTEMIC SYMPTOMS**	

Number of individuals considered for analysis of systemic symptoms (n)	7809	3189
One or more systemic symptom (n (%))	1104 (14·1)	266 (8·3)
Headache (n (%))	291 (3·7)	63 (2·0)
Fatigue (n (%))	257 (3·3)	57 (1·8)
Sore throat (n (%))	272 (3·5)	54 (1·7)
Rhinorrhoea (n (%))	294 (3·8)	48 (1·5)
Persistent cough (n (%))	116 (1·5)	30 (0·9)
Anosmia/Dysosmia (n (%))	110 (1·4)	30 (0·9)
Sneezing (n (%))	169 (2·2)	28 (0·9)
Chills & Shivers (n (%))	104 (1·3)	25 (0·8)
Nausea (n (%))	80 (1·0)	21 (0·7)
Dizziness (n (%))	100 (1·3)	20 (0·6)
Fever (n (%))	121 (1·5)	19 (0·6)
Abdominal pain (n (%))	73 (0·9)	15 (0·5)
Hoarse voice (n (%))	58 (0·7)	15 (0·5)
Lymphadenopathy (n (%))	30 (0·4)	14 (0·4)
Myalgia (n (%))	57 (0·7)	11 (0·3)
Arthralgia (n (%))	62 (0·8)	9 (0·3)
Low appetite (anorexia) (n (%))	52 (0·7)	8 (0·3)
Dyspnoea (n (%))	27 (0·3)	8 (0·3)
Earache (n (%))	26 (0·3)	8 (0·3)
Low mood (depression) (n (%))	25 (0·3)	7 (0·2)
Diarrhoea (n (%))	29 (0·4)	6 (0·2)
Brain Fog (n (%))	31 (0·4)	6 (0·2)
Delirium (n (%))	30 (0·4)	6 (0·2)
Chest pain (n (%))	27 (0·3)	4 (0·1)
Palpitations (n (%))	8 (0·1)	4 (0·1)
Hair loss (n (%))	1 (<0·1)	4 (0·1)
Tinnitus (n (%))	18 (0·2)	3 (0·1)

	**CUTANEOUS SYMPTOMS**	

Eye Soreness	35 (0·4)	8 (0·3)
Skin Burning (n (%))	14 (0·2)	3 (0·1)
Rash (n (%))	5 (0·1)	2 (0·1)
Red welts on lips (n (%))	8 (0·1)	1 (<0·1)
Blisters on feet	2 (<0·1)	0 (0·0)

	**LOCAL SYMPTOMS**	

Number of individuals considered for analysis of local symptoms	7751	2876
One or more local symptom (n (%))	5485 (70·8)	1731 (60·2)
Tenderness (n (%))	3557 (45·9)	1183 (41·1)
Pain (n (%))	3240 (41·8)	954 (33·2)
Warmth (n (%))	378 (4·9)	128 (4·5)
Swelling (n (%))	388 (5·0)	90 (3·1)
Redness (n (%))	298 (3·8)	71 (2·5)
Axillary Lymphadenopathy (n (%))	114 (1·5)	20 (0·7)
Pruritis (n (%))	88 (1·1)	20 (0·7)
Number of individuals presenting to hospital post-vaccination*(n (%))	6 of 7809 (0·1)	0 of 3189 (0·0)

BMI is body mass index; SD is standard deviation.

⁎ Presentation to hospital: presenting to the emergency department and/or admission to hospital. Reason for presentation unknown.

**Figure 6:**
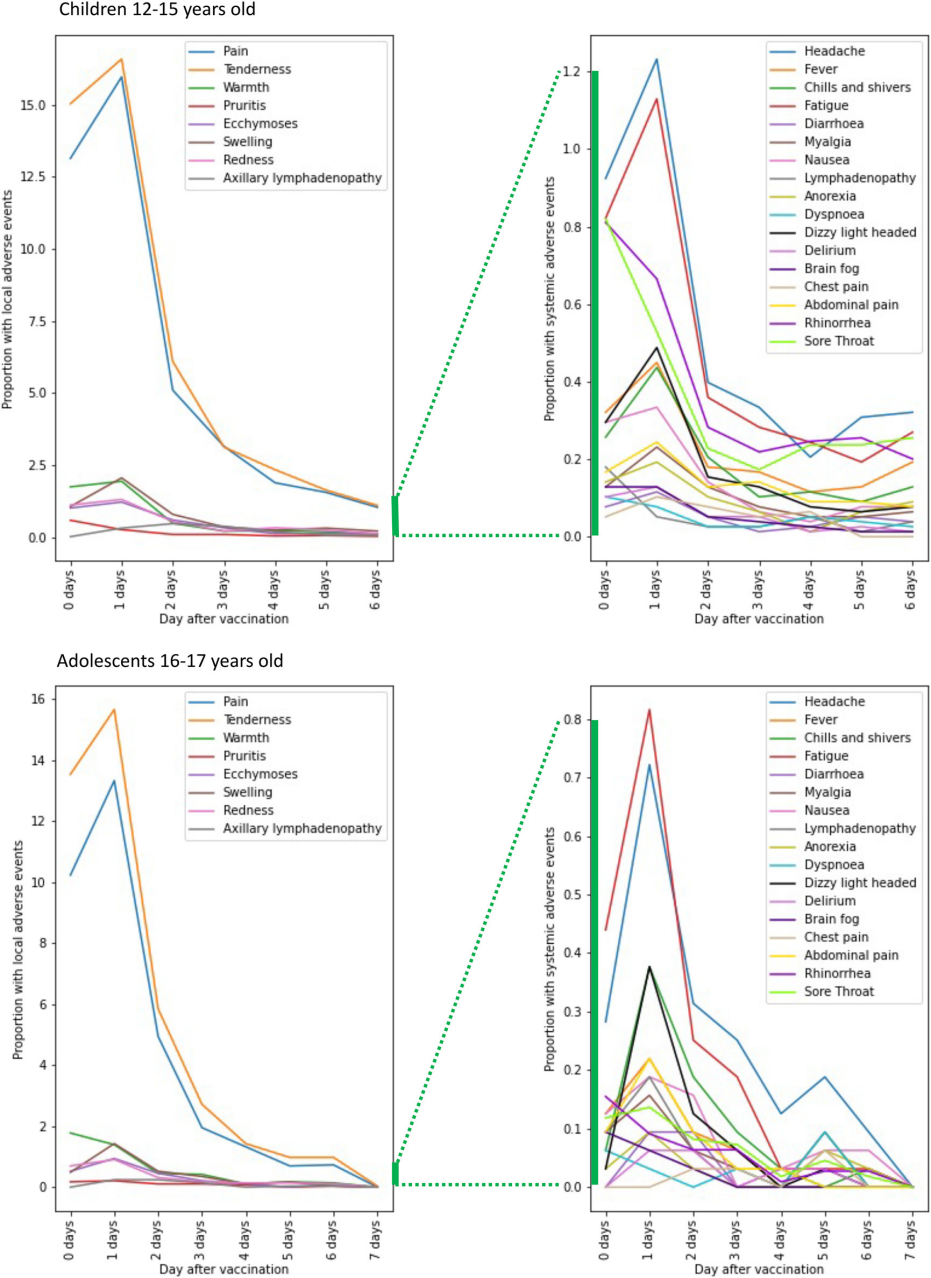
Proportion of CA aged 12 to 17 years old, separated by age group, reporting vaccine side-effects after one dose of BNT162b2. Left panels: local symptoms at the arm of injection within 7 days; right panels: systemic symptoms presenting within the same 7 days. Data for children aged 12-15 years are shown in upper panels and for adolescents aged 16-17 years in lower panels.

Among children, 5485 (70·8%) of 7751 had one or more local symptom post-vaccination, most commonly tenderness (3557 (45·9%) of 7751 children) and local pain at the injection site (3240 (41·8%)). 1104 (14·1%) of 7809 children had one or more systemic symptom, most commonly rhinorrhoea (294 (3·8%) of 7809 children), headache (291 (3·7%), sore throat (272 (3·5%)), and fatigue (257 (3·3%)). Cutaneous symptoms were rare (<0·5%). Post-vaccination symptoms (both local and systemic) usually occurred on the first day post-vaccination, and resolved quickly in most children (3 days in most cases) (Figure 6, upper panel).

Among adolescents, 1731 (60·2%) of 2876 had one or more local symptom post-vaccination, again most commonly tenderness (1183 (41·1%) of 2876 adolescents) and local pain (954 (33·2%)). 266 (8·3%) of 3189 had one or more systemic symptom, most commonly headache (63 (2·0%) of 3189 adolescents), fatigue (57 (1·8%)), and sore throat (54 (1·7%)). Cutaneous symptoms were again rare (<0·4%). Similarly, both systemic and local symptoms usually occurred on the first day post-vaccination and resolved quickly (∼3 days in most cases) (Figure 6, lower panel).

Supplementary Figure 7 shows post-vaccination local and systemic symptom profiles for the cohort overall.

Six (0·1%) of 7809 children and none of the 3189 adolescents presented to hospital after vaccination.

No question was asked specifically regarding myocarditis or pericarditis. Chest pain was reported in 27 (0.3%) of 7809 children, and 4 (0.1%) of 3189 adolescents; and dyspnoea in 27 (0.3%) of 7809 children and 8 (0.3%) of 3189 adolescents. Free-text scrutiny revealed five cases of tingling of the arm, hand, or fingers at the side of injection; two cases of insomnia; and one of lethargy. No reports were found of anaphylaxis, thrombosis, cytopenia, myocarditis, pericarditis, Guillain-Barré syndrome, or multi-organ failure. Analogously, we found no terms suggesting medical tests related to myocarditis or pericarditis, such as electrocardiography, echography, or creatinine kinase tests.

## Discussion

Here we show that vaccination with a single dose of BNT162b2 reduced risk of SARS-CoV-2 infection in CA aged 12-17 years, at least over the time frame of this study. The protective effect of vaccination was evident early, with risk of infection reduced 14 days post-vaccination and prompt divergence of Kaplan-Meier curves for infection-free survival. However, vaccination effectiveness may vary according to prevalent SARS-CoV-2 variants, and appeared greater for Delta than Omicron. Vaccinated CA with prior SARS-CoV-2 infection had near-total protection against infection with the Delta variant, with re-infection close to null for at least 100 days; currently insufficient data are available to make similar comment regarding the Omicron variant. However, >90% of children in UK are currently thought to have had SARS-CoV-2 infection (modelled antibody prevalence from December 7, 2020 to February 4, 2022 of 82% in children aged 8-11 years and 94% in children aged 12-15 years; https://www.ons.gov.uk/peoplepopulationandcommunity/healthandsocialcare/conditionsanddiseases/bulletins/coronaviruscovid19infectionsurveyantibodyandvaccinationdatafortheuk/23february2022); thus our finding here may inform discussions regarding two-dose vaccination policy, particularly for CA with prior infection.

Our finding of a strong and sustained effect of prior SARS-CoV-2 infection on post-vaccination effectiveness concurs with other studies also showing an effect of prior infection upon immunity. In one such, 32 participants with prior SARS-CoV-2 infection were assessed post-vaccination and evidenced strong neutralising antibody responses after first mRNA vaccination that did not increase further following a second dose and was higher than uninfected participants who had received two vaccine doses (*n* = 139).^19^

Our community data regarding effectiveness of single-dose vaccination (at least for the considered time-scale) adds to previous publications. Phase 3 randomised trials in persons aged 12 years and older demonstrated high two-dose vaccine efficacy (95% CI, 75.3 to 100%^6^) with immunogenicity against earlier SARS-CoV-2 wild-type and Alpha variants^6^^,^^7^ and protection against severe COVID-19.^11^^,^^20^ The PROTECT trial, which included 243 participants aged 12-17 years, estimated adjusted vaccination effectiveness of 94% (83%-98%) after two doses of BNT162b2.^21^ Community data on vaccination effectiveness in adolescents are limited.^22^^,^^23^ A national study in Israel observed vaccine effectiveness of 87.1%−91.2%, from two weeks after administration of second vaccine dose; and, considered until end of August 2021, none of the vaccinated and 33 of 9969 unvaccinated adolescents who tested positive for SARS-CoV-2 (0.33%) were hospitalised for severe illness.^24^

Our data suggesting a variant-dependent difference in vaccination effectiveness concords with a recent pre-printed study in England,^25^ that used a test-negative case-control design to assess PCR-confirmed COVID-19. This study showed two-dose BNT162b2 vaccination in 12- to 17-year-old CA was less effective against symptomatic disease from Omicron vs. Delta SARS-CoV-2 variants (variant genotype assumed from prevalence in population), with vaccination effectiveness against COVID-19 peaking 14 days after first dose for Delta and Omicron at 75-76%% and 50-51%% respectively, and decreasing to 36-48% and 23-27% by 8 weeks.

Vaccination also resulted in a modest change in COVID-19 profile. Vaccinated CA with post-vaccination SARS-CoV-2 infection appeared to have milder disease (i.e., fewer symptoms) than unvaccinated CA, at least during the time of Delta predominance. Less difference was evident during Omicron predominance, especially in adolescents, noting here that disease appeared milder during Omicron predominance compared with Delta predominance, in both vaccinated and unvaccinated CA. Relevantly, our data in unvaccinated CA are consistent with our earlier study,^3^ and that of others (https://www.ons.gov.uk/peoplepopulationandcommunity/healthandsocialcare/conditionsanddiseases/articles/coronaviruscovid19latestinsights/Overview#age), in that COVID-19 in children is usually milder, and of shorter duration, compared with adults,^26^ with much lower risk of severe disease, death and prolonged symptoms. Thus, *a priori* the capacity to show a large impact of vaccination on disease profile in CA (in contrast to adults) was limited. Nonetheless, even during Omicron predominance odds ratios showed several symptoms were significantly less common in vaccinated vs. unvaccinated children. Our descriptive data of hospital presentations showed slightly lower numbers in vaccinated vs. unvaccinated children (0·64% vs. 1·58% for Omicron) but the opposite finding in adolescents (2·13% vs. 1·50% for Omicron). No robust comparisons can be made here.

Adverse events suggestively attributable to vaccination were common but dominated by local symptoms (70·8% of children, 60·2% of adolescents) which resolved rapidly in most cases. Our data here are very similar to those of the v-safe study (60%), a USA smartphone-based surveillance study of adolescents aged 12-17 years (N = 129,059) who completed at least one health check-in survey within one week after receiving a first dose of BNT162b2 vaccine.^10^ Systemic side-effects in our cohort were uncommon, non-critical, and non-persistent. The profile of systemic side-effects we observed was similar to that recorded in the pivotal clinical trial for the BNT162b2 vaccine^6^ but much less common (e.g., for headache: 2·0%-3·7% in our cohort vs. 55·0-65·0% in the pivotal trial; for fatigue: 1·8%-3·3% vs. 60·0-66·0%). In the v-safe study, headaches were reported in 25-30%, and fatigue in 27-34%.^10^ It may be that only more severe systemic side effects were proxy-reported in our study. However, the nocebo effect may also be relevant in explaining the discrepancy (i.e., that individuals warned of potential side-effects may report them even when receiving the placebo), with a recent meta-analysis reporting extremely common nocebo responses in trials of COVID-19 vaccines (76.0% of systemic effects after first dose and 51.8% after second dose).^27^

The strengths of our study include community, real-time data generated from thousands of app users and a stringent study design. However, we also acknowledge the weaknesses of our study. Our data are generated by a volunteer citizen-science initiative delivered through an app, with the CSS cohort more likely to be of White Caucasian ethnicity, higher family socioeconomical status, and with a higher representation of healthcare workers than the overall UK population.^14^ By virtue of UK government policy timing and age-tiered approach, our study was restricted to considering single-dose vaccination with BNT162b2 in CA aged 12-17 years; our results may not apply to other vaccines or in younger age-groups. Our study was conducted with the prevailing SARS-CoV-2 variants Delta and Omicron; the impact of vaccination (both effectiveness and durability) and the profile of post-vaccination COVID-19 might differ with other variants. Indeed, the durability of vaccine protection against Omicron beyond 3 months remains to be determined in our cohort, due to insufficient data. The ability to assess vaccination effectiveness in CA naÿve to SARS-CoV-2 is dwindling rapidly, due to widespread infection in the community, and particularly in this age tier. Vaccinated CA were also much more likely to be tested for SARS-CoV-2 than unvaccinated CA, for reasons that we are unable to determine. Our data do not allow determination of causal variants for CA testing positive either post- or prior to vaccination: we can only infer likely variant based on population prevalence at time of testing. Vaccination can attenuate viral RNA shedding;^28^ this might result in the overestimation of vaccination effectiveness by reducing viral detection among infected individuals who received vaccination. In addition, divergence of infection risk soon after vaccination may indicate that the vaccinated had a lower risk of infection than the unvaccinated. Pertinently here, we could not incorporate into our analysis behaviours that affect risk of infection, such as mask-wearing, distancing, quarantines, and school attendance. Importantly, although myocarditis and pericarditis have been reported as significant adverse effects in younger populations,^9^ no dedicated question assessed this specifically; however, our data regarding chest pain and dyspnoea were assessed and free text reports scrutinised for such adverse diagnoses without showing any signal. Lastly, calculation of the burden of disease was based on the number of symptoms in this study, and did not include a measure of their intensity.

Importantly, this study should be regarded as an interim report of the effects of one-dose BNT162b2 vaccine. Pivotal clinical trials,^6^^,^^7^ product licensing, and most national recommendations indicate that more comprehensive immunization is achieved after at least two time-spaced doses of BNT162b2 or other SARS-CoV-2 vaccinations. However, our data suggest some nuance may be appropriate regarding recommendations for second-dose vaccination, considered temporally (particularly with reference to prior infection) and with respect to new variant emergence.

## Conclusions

One dose of BNT162b2 vaccine provides protection against SARS-CoV-2 infection in CA aged 12-17 years. Vaccine protection is modulated by the SARS-CoV-2 variant type (with lower protection for Omicron compared with Delta varaints), and by previously-acquired infection-induced immunity. Overall, BNT162b2 vaccine is well tolerated in children and adolescents, with common but fast-fading local effects, and uncommon and transient systemic effects. Severity of COVID-19 presentation after one dose of vaccine is generally milder, although unvaccinated CA generally have an uncomplicated course too. We contribute data for the benefit of governments and public health agencies, to be considered within the debate on vaccination in children, and for designing public health strategies to control future developments of the COVID-19 pandemic.

## Contributors

Conceptualisation: EM, LCa, CJS, SO, MAb, ELD

Data Curation: LCa, JD, MAn

Data Collection System including Software and Resources: BM, LCh, JCP, LP, AM, JW, EK, TJS, CHS, MM, SO, CHS

Formal Analyses: EM, LCa, KK

Funding Acquisition: SO, TDS, CJS

Project Supervision and Coordination: EM, ELD Validation: EM, LCa, JD, MAn, ELD. All authors had access to the COVID Symptom Study dataset, which is also accessible to researchers in the public interest.

Writing – original draft: EM, LCa, MAb, ELD

Writing: Review & Editing: All authors

## Data sharing statement

Data collected in the COVID Symptom Study smartphone application may be shared with other health researchers through the UK National Health Service-funded Health Data Research UK (HDRUK) and Secure Anonymised Information Linkage consortium, housed in the UK Secure Research Platform (Swansea, UK). Anonymised data are available to be shared with researchers according to their protocols in the public interest (https://web.www.healthdatagateway.org/dataset/fddcb382-3051-4394-8436-b92295f14259).

## Declaration of interests

JCP, LP, AM, JW are employees of Zoe Limited.

TDS and SO report being consultants for Zoe Limited, during the conduct of the study.

All other authors have nothing to declare.

## Appendix Supplementary materials

Image, application 1
