# Supplementary material for: Post-vaccination infection rates and modification of COVID-19 symptoms in vaccinated UK school-aged children and adolescents: A prospective longitudinal cohort study
Source: Lancet Reg Health Eur. 2022 Jul 8;19:100429. doi: 10.1016/j.lanepe.2022.100429 (PMC9263281; doi:10.1016/j.lanepe.2022.100429)
Supplement: Supplementary file 1 [file mmc1.docx]

**SUPPLEMENTARY DOCUMENT**

**Vaccination against SARS-CoV-2 in UK school-aged children and adolescents decreases infection rates and reduces COVID-19 symptoms.**

Erika Molteni PhD^1^, Liane S. Canas PhD^1^, Kerstin Kläser PhD^1^, Jie Deng PhD^1^, Sunil S. Bhopal PhD^2^, Robert C. Hughes MPH^3^, Liyuan Chen MSc^1^, Benjamin Murray MSc^1^, Eric Kerfoot PhD^1^, Michela Antonelli PhD^1^, Carole H. Sudre PhD^1^, Joan Capdevila Pujol PhD^4^, Lorenzo Polidori MSc^4^, Anna May MSc^4^, Prof Alexander Hammers PhD^1^, Jonathan Wolf MSc^4^, Prof Tim D. Spector PhD^5^, Claire J. Steves PhD^5,6^, Prof Sebastien Ourselin PhD^1^, Michael Absoud PhD*^,7,8^, Marc Modat PhD*^,1^, Prof Emma L. Duncan PhD*^,5,9^.

*Equal senior author

**Supplementary Table 1. List of symptom questions asked by the COVID Symptom Study application during the current study period**.

| **Symptom** | **COVID Symptom Study app question** |
| --- | --- |
| Fever | Fever (at least 37.8C or 100F) |
| Persistent Cough | Persistent cough (coughing a lot for more than an hour or 3 or more coughing episodes in 24 hours) |
| Fatigue | Unusual fatigue (no; mild fatigue; severe fatigue/ I struggle to get out of bed) |
| Dyspnoea | Shortness of breath or trouble breathing (no; yes mild symptoms/ slight shortness of breath during ordinary activity: yes significant symptoms/ breathing is comfortable only at rest; yes, severe symptoms/ breathing is difficult even at rest). |
| Anosmia/Ageusia | Loss of smell / taste |
| Hoarse Voice | Unusually hoarse voice |
| Chest Pain | Unusual chest pain or tightness in your chest |
| Abdominal Pain | Unusual abdominal pain or stomach-ache |
| Diarrhoea | Diarrhoea |
| Delirium | Confusion, disorientation or drowsiness |
| Eye Soreness | Do your eyes have any unusual eye-soreness or discomfort (e.g. light sensitivity, excessive tears, or pink/red eye)? |
| Low appetite (anorexia) | Skipping meals |
| Headache | Headache |
| Nausea | Nausea or vomiting |
| Dizziness | Dizziness or light-headedness |
| Sore Throat | Sore or painful throat |
| Myalgias | Unusual strong muscle pains or aches |
| Red Welts | Raised, red, itchy welts on the skin or sudden swelling of the face or lips |
| Blisters | Red/purple sores or blisters on your feet, including your toes |
| Rashes | Rash on your arms or torso |
| Sensitive Skin | Strange, unpleasant sensations in your skin like pins & needles or burning |
| Hair Loss | Unusual hair loss |
| Low mood (depression) | Feeling down, depressed, or hopeless |
| Brain Fog | Loss of concentration or memory (brain fog) |
| Dysosmia/Dysgeusia | Altered smell / taste (things smell or taste different to usual) |
| Rhinorrhoea | Runny nose |
| Sneezing | Sneezing more than usual |
| Ear Pain | Earache |
| Tinnitus | Ringing in your ears |
| Lymphadenopathy | Swollen neck glands |
| Palpitations | Unusually fast or irregular heartbeat (palpitations) |
| Arthralgias | Unusual joint pains or aches |
| Mouth Ulcers | Mouth or tongue ulcers |
| Tongue Changes | Changes to tongue surface |

**Supplementary Table 2. List of questions about local symptoms at the site of vaccination, asked by the COVID Symptom Study application during the current study period**.

| **Symptom** | **Question asked through the app:** Are you experiencing any symptoms near the injection site? (Check all that apply). |
| --- | --- |
| **Pain** | Pain |
| **Redness** | Redness |
| **Swelling** | Swelling |
| **Axillary lymphadenopathy** | Swollen glands in the armpit |
| **Warmth** | Warmth |
| **Pruritis** | Itch |
| **Tenderness** | Tenderness |
| **Ecchymoses** | Bruising |
| **Other** | Other: (free text) |

**Supplementary Table 3. Cohort selection criteria for each analysis and associated sample sizes.**

| **Selection criteria** | 12-15-year-old group  (children) | 16-17-year-old group  (adolescents) |
| --- | --- | --- |
| **Post-vaccination infection risk.**  ***Vaccinated:***   - One dose of BNT162b2 vaccination. - Absence of documented infection prior to vaccination. - SARS-CoV-2 test at least 14 days after one dose of BNT162b2 vaccination. - First SARS-CoV-2 positive test (if any), and any negative test before the first positive.   ***Unvaccinated:***   - First SARS-CoV-2 positive test (if any), and any negative test before the first positive. | Delta=5,658  Omicron=6,416  Delta=3,977  Omicron=723 | Delta=4,857  Omicron=857  Delta=816  Omicron=207 |
| **Infection over time:**  ***- Vaccinated CA without prior SARS-CoV-2 infection:***   - One dose of BNT162b2 vaccination. - Absence of documented infection prior to vaccination. - SARS-CoV-2 test any time after one dose of BNT162b2 vaccination. - First SARS-CoV-2 positive test (if any), and any negative test before the first positive.   ***- Unvaccinated (with tests matched to vaccinated CA without prior infection 1:1 by week of test):***  - First SARS-CoV-2 positive test (if any), and any negative test before the first positive.  ***- Vaccinated CA with prior SARS-CoV-2 infection matched to vaccinated CA without prior SARS-CoV-2 infection 1:1 by week of test):***   - One dose of BNT162b2 vaccination. - Documented positive test result prior to vaccination. - Further positive SARS-CoV-2 test any time after one dose of BNT162b2 vaccination. - First SARS-CoV-2 positive test (if any), and any negative test before the first positive.   ***- Vaccinated without prior infection (with tests matched to vaccinated previously infected CA, matched 1:1 by week of test, then age and gender):***  - First SARS-CoV-2 positive test (if any), and any negative test before the first positive. | Delta=4,240  Omicron=1,339  Delta=11,156  Omicron=1,339  Delta=479  Omicron=717  Delta=479  Omicron=710 | Delta=9,048  Omicron=262  Delta=2,132  Omicron=262  Delta=963  Omicron=22  Delta=963  Omicron=29 |
| **Post-vaccination infection profile (including hospital presentation)**  Populations matched* by age, gender week of test and BMI.  ***Vaccinated:***   - One dose of BNT162b2 vaccination. - Absence of documented infection prior to vaccination. - SARS-CoV-2 test at least 14 days after one dose of BNT162b2 vaccination. - First SARS-CoV-2 positive test.   ***Unvaccinated:***   - First SARS-CoV-2 positive test   * Matching performed using Euclidean distance based on the variables mentioned above. 1:1 matched population not performed. | Delta=320  Omicron=1,269  Delta=320  Omicron=409 | Delta=564  Omicron=166  Delta=564  Omicron=97 |
| **Local and systemic symptoms attributable to vaccination**   - One dose of BNT162b2 vaccination. - Absence of documented infection prior to vaccination. - At least one report of symptoms (including their absence) within 7 days after vaccine uptake. | Systemic Symptoms = 7,809  Local Symptoms = 7,751 | Systemic Symptoms= 3,189  Local Symptoms =  2,876 |

**Supplementary Table 4. Odds ratios for symptom prevalence in vaccinated vs. unvaccinated CA, first testing positive for SARS-CoV-2 during periods of Delta (left) and Omicron (right) variant predominance.**

Asymptomatic CA testing positive for SARS-CoV-2 are included in the computation.

Bold encodes significant p-values after false discovery rate correction (alpha =0.05).

|  | **CA: 12-15 yo** | | | | **CA: 16-17 yo** | | | |
| --- | --- | --- | --- | --- | --- | --- | --- | --- |
|  | **Delta** | | **Omicron** | | **Delta** | | **Omicron** | |
| **Symptoms** | **OR [IQR]** | **p-value** | **OR [IQR]** | **p-value** | **OR [IQR]** | **p-value** | **OR [IQR]** | **p-value** |
| Abdominal pain | 0·71 [0·43; 1·17] | 0·1770 | **0·49 [0·35; 0·70]** | **0·0001** | **0·50 [0·33; 0·77]** | **0·0015** | 0·57 [0·21; 1·57] | 0·2791 |
| Dysosmia | 0·67 [0·44; 1·00] | 0·0501 | **0·59 [0·41; 0·85]** | **0·0044** | 0·82 [0·62; 1·09] | 0·1637 | 1·31 [0·50; 3·43] | 0·5878 |
| Blisters on feet | 0·33 [0·03; 3·20] | 0·3371 | 0·62 [0·11; 3·43] | 0·5830 | 0·24 [0·03; 2·01] | 0·1876 | 0 [0; inf] | 0·9993 |
| Brain Fog | 0·76 [0·40; 1·43] | 0·3937 | 0·59 [0·38; 0·91] | 0·0175 | 0·76 [0·53; 1·10] | 0·1454 | 0·73 [0·26; 2·10] | 0·5622 |
| Chest Pain | 0·72 [0·36; 1·43] | 0·3489 | 0·77 [0·48; 1·24] | 0·2867 | **0·54 [0·36; 0·82]** | **0·0040** | 0·65 [0·21; 2·08] | 0·4718 |
| Chills or Shivers | 0·63 [0·42; 0·93] | 0·0217 | 0·75 [0·56; 1·00] | 0·0481 | 0·75 [0·56; 0·99] | 0·0455 | 0·68 [0·34; 1·33] | 0·2573 |
| Confusion | **0·22 [0·07; 0·67]** | **0·0073** | **0·46 [0·26; 0·82]** | **0·0079** | 0·67 [0·43; 1·04] | 0·0724 | 0·44 [0·14; 1·33] | 0·1453 |
| Diarrhoea | 0·44 [0·22; 0·89] | 0·0231 | 0·81 [0·47; 1·39] | 0·4451 | **0·55 [0·34; 0·88]** | **0·0128** | 0·98 [0·36; 2·73] | 0·9749 |
| Dizziness | **0·48 [0·31; 0·73]** | **0·0008** | **0·52 [0·39; 0·70]** | **<0·0001** | **0·59 [0·43; 0·81]** | **0·0010** | 0·43 [0·22; 0·86] | 0·0173 |
| Tinnitus | 0·72 [0·24; 2·11] | 0·5481 | 0·77 [0·44; 1·36] | 0·3736 | 0·60 [0·33; 1·08] | 0·0896 | 0·47[0·13; 1·72] | 0·2519 |
| Earache | 0·48 [0·23; 1·01] | 0·0536 | 0·60 [0·38; 0·95] | 0·0305 | 0·70 [0·44; 1·10] | 0·1240 | 0·33 [0·11; 0·99] | 0·0477 |
| Eyes soreness | **0·39 [0·24; 0·64]** | **0·0002** | **0·62 [0·43; 0·88]** | **0·0082** | **0·49 [0·36; 0·69]** | **<0·0001** | 0·73 [0·31; 1·69] | 0·4559 |
| Fatigue | **0·66 [0·48; 0·90]** | **0·0097** | 0·83 [0·65; 1·03] | 0·0868 | **0·64 [0·50; 0·82]** | **0·0003** | 0·66 [0·39; 1·12] | 0·1221 |
| Low Mood | 0·64 [0·33; 1·24] | 0·1832 | 0·65 [0·39; 1·08] | 0·0962 | **0·61 [0·40; 0·94]** | **0·0230** | 1·03 [0·32; 3·35] | 0·9611 |
| Fever | 0·71 [0·49; 1·02] | 0·0642 | **0·67 [0·52; 0·87]** | **0·0029** | **0·56 [0·42; 0·73]** | **<0·0001** | 0·78 [0·41; 1·50] | 0·4623 |
| Hair Loss | 0·95 [0·06; 15·66] | 0·9699 | 1·24 [0·13; 11·59] | 0·8492 | 0·78 [0·28; 2·18] | 0·6394 | **1 [1;1]** | **0** |
| Headache | **0·55 [0·40; 0·75]** | **0·0002** | **0·76 [0·60; 0·95]** | **0·0148** | **0·61 [0·48; 0·78]** | **0·0001** | 0·76 [0·45; 1·29] | 0·3169 |
| Hoarse voice | 0·74 [0·45; 1·20] | 0·2206 | 1·12 [0·79; 1·60] | 0·5343 | **0·61 [0·44; 0·84]** | **0·0026** | 1·14 [0·48; 2·70] | 0·7671 |
| Palpitations | 0·72 [0·23; 2·29] | 0·5758 | 1·09 [0·46; 2·57] | 0·8459 | 0·74 [0·37; 1·47] | 0·3844 | 2·08 [0·22; 19·74] | 0·5246 |
| Anosmia | 0·79 [0·54; 1·16] | 0·2213 | **0·48 [0·31; 0·73]** | **0·0007** | 0·81 [0·62; 1·05] | 0·1138 | 0·53 [0·20; 1·36] | 0·1852 |
| Nausea | **0·35 [0·20; 0·59]** | **0·0001** | 0·72 [0·50; 1·02] | 0·0652 | **0·51 [0·35; 0·74]** | **0·0004** | 0·54 [0·23; 1·27] | 0·1588 |
| Persistent Cough | 0·74 [0·52; 1·05] | 0·0873 | 1·19 [0·90; 1·59] | 0·2264 | **0·73 [0·57; 0·94]** | **0·0140** | 1·79 [0·87; 3·67] | 0·1132 |
| Rashes | 0·36 [0·10; 1·39] | 0·1394 | 0·66 [0·29; 1·51] | 0·3296 | **0·27 [0·11; 0·72]** | **0·0080** | 0 [0; inf] | 0·9992 |
| Red Welts FL | 1·00 [0·14; 7·14] | 0·9989 | 0·64 [0·26; 1·62] | 0·3507 | 0·43 [0·19; 0·98] | 0·0443 | 1·10 [0·16; 7·58] | 0·9226 |
| Rhinorrhoea | **1·54 [1·13; 2·11]** | **0·0069** | 0·96 [0·76; 1·20] | 0·7035 | 1·29 [1·02; 1·64] | 0·0347 | 0·92 [0·53; 1·60] | 0·7692 |
| Dyspnoea | 0·81 [0·43; 1·52] | 0·5043 | 0·97 [0·59; 1·58] | 0·8868 | **0·65 [0·44; 0·96]** | **0·0289** | 1·21 [0·42; 3·46] | 0·7287 |
| Sensitive Skin | 0·53 [0·19; 1·46] | 0·2215 | 0·68 [0·35; 1·30] | 0·2439 | **0·43[0·24; 0·77]** | **0·0043** | 0·13 [0·03; 0·67] | 0·0143 |
| Low Appetite | **0·47 [0·29; 0·76]** | **0·0018** | **0·54 [0·39; 0·75]** | **0·0002** | **0·40 [0·28; 0·57]** | **0·0000** | 0·71 [0·33; 1·54] | 0·3855 |
| Sneezing | 1·12 [0·79; 1·59] | 0·5174 | 0·91 [0·70; 1·18] | 0·4751 | **1·28 [1·00; 1·67]** | **0·0609** | 1·26 [0·68; 2·34] | 0·4551 |
| Sore throat | 0·85 [0·62; 1·17] | 0·3161 | 0·92 [0·74; 1·16] | 0·4899 | **0·62 [0·49; 0·79]** | **0·0001** | 0·77 [0·45; 1·29] | 0·3152 |
| Lymphadenopathy | 0·65 [0·34; 1·24] | 0·1901 | 0·77 [0·48; 1·24] | 0·2849 | **0·38 [0·24; 0·61]** | **0·0001** | 1·01 [0·39; 2·61] | 0·9821 |
| Myalgias | **0·49 [0·29; 0·84]** | **0·0099** | **0·48 [0·34; 0·68]** | **< 0.0001** | **0·50 [0·35; 0·73]** | **0·0003** | 0·56 [0·27; 1·18] | 0·1282 |
| Arthralgias | **0·33 [0·19; 0·56]** | **0·0000** | 0·72 [0·52; 1·00] | 0·0474 | **0·58 [0·42; 0·80]** | **0·0010** | 0·39 [0·20; 0·76] | 0·0057 |

**Supplementary Material 1. Proxy-reported post-vaccination infection risk in CA without prior SARS-CoV-2 infection.**

To assess real-world effectiveness of one-dose vaccination, vaccinated and unvaccinated CA naïve to SARS-CoV-2 were compared. To allow time for induction of immunity ^1^, test results for vaccinated CA were considered from 14 days after vaccination, as previously^2^. Unvaccinated CA were excluded if they had tested positive before 5 August 2021 and were only considered until the first positive test result was recorded, if such existed.

For each vaccinated or unvaccinated CA, all negative tests until the first positive test (included, if such existed) were considered; after the first positive test, all subsequent tests were ignored. As previously ^2^, test results were grouped by week: for CA with at least one negative and one positive test within a single week, the positive test was used. In case of multiple negative tests within one week, the last one was used.

To control for viral prevalence and strain, vaccinated CA were matched with the unvaccinated by week of testing. As previously ^2^, an unvaccinated CA could serve as a control for more than one week, if tested within each corresponding week.

We used Poisson regression to compare weekly incidence of SARS-CoV-2 infection in the vaccinated and unvaccinated CA, adjusting for number of tests, age, number of co-morbidities, sex, and weekly incidence of infection nationally (by controlling for week of testing through a categorical variable). We obtained the adjusted risk reduction as RR=(riskratio_n_−1)*100, where n encodes the week considered and *riskratio* is the ratio of infection rates in vaccinated vs. unvaccinated individuals, estimated through the Poisson model. Descriptive statistics are also presented below.

**References**

1 Dowell AC, Butler MS, Jinks E, *et al.* Children develop strong and sustained cross-reactive immune responses against Spike protein following SARS-CoV-2 infection, with enhanced recognition of variants of concern. *medRxiv* 2021.

2 Menni C, Klaser K, May A, *et al.* Vaccine side-effects and SARS-CoV-2 infection after vaccination in users of the COVID Symptom Study app in the UK: a prospective observational study. *The Lancet Infectious Diseases* 2021; **21**. DOI:10.1016/S1473-3099(21)00224-3.

**Supplementary Figure 1. Infection risk reduction after single vaccination with BNT162b2 in 12- to17-year-old CA during periods of Delta (left) and Omicron (right) variant predominance in UK.**

Bars represent risk reduction at 14-30 days, 1-2 months, and 2-3 months for post-vaccination infection, compared with unvaccinated CA. The black lines show 95% CIs. Number of observations (i.e., tests) of CA aged 12-17 years:

for period of Delta variant predominance n (test) =15,308 (4,793 unvaccinated, 10,515 vaccinated);

for period of Omicron variant predominance n (test) =8,203 (930 unvaccinated, 7,273 vaccinated).

**Supplementary Figure 2. Infection risk reduction after first dose BNT162b2 vaccination in adolescents aged 16-17 years, during periods of Delta (left) and Omicron (right) variant predominance in UK.**

Bars represent risk reduction at 14-30 days, 1-2 months, and 2-3 months in adolescents post-vaccination, compared with unvaccinated adolescents. Black lines represent 95% CIs. Number of observations (i.e., tests) of adolescents: during Delta variant predominance in UK: n(test) = 5,673 (816 unvaccinated, 4,857 vaccinated); during Omicron variant predominance: n(test) = 1,064 (207 unvaccinated, 857 vaccinated).

**Supplementary Figure 3. Infection risk reduction after first dose BNT162b2 vaccination in children aged 12-15 years, during periods of Delta (left) and Omicron (right) variant predominance in UK.**

Bars represent risk reduction at 14-30 days, 1-2 months, and 2-3 months for post-vaccination infection, compared with unvaccinated children. The black lines show 95% CIs. Number of observations (i.e., tests) of children:

for Delta variant predominance, n(test) = 9,635 (3,977 unvaccinated, 5,658 vaccinated); for Omicron variant predominance, n(test) = 7,139 (723 unvaccinated, 6,416 vaccinated).

**Supplementary Figure 4. Symptom duration in vaccinated and unvaccinated CA with first symptomatic SARS-CoV-2 infection during periods of Delta (left panels) and Omicron (right panels) variant predominance** Asymptomatic CA testing positive for SARS-CoV-2 are excluded from computation of symptom duration. Bars show median duration; black lines show IQR.

**Supplementary Figure 5. Persistence of individual symptoms over 7 days after first testing positive for SARS-CoV-2, in vaccinated (left) and unvaccinated (right) adolescents aged 16- 17 years, during periods of Delta (top) and Omicron (bottom) variant prevalence in UK.**

Each row represents persistence over time (in days) of each symptom for individuals reporting that symptom. Day 0 is defined as the first day of symptom presentation.

**Supplementary Figure 6. Persistence of individual symptoms over 7 days after first testing positive for SARS-CoV-2, in vaccinated (left) and unvaccinated (right) children aged 12-15 years old, during periods of Delta (top) and Omicron (bottom) variant prevalence in UK.**

Each row represents persistence over time (in days) of each symptom for individuals reporting that symptom. Day 0 is defined as the first day of symptom presentation.

**Supplementary Figure 7. Proportion of CA aged 12 to 17 years reporting vaccine side-effects after one dose BNT162b2.**

Local effects at the arm of injection considered for 7 days are presented in the left panel.

Systemic symptoms presenting within the same 7 days after vaccination are presented in the right panel.

**
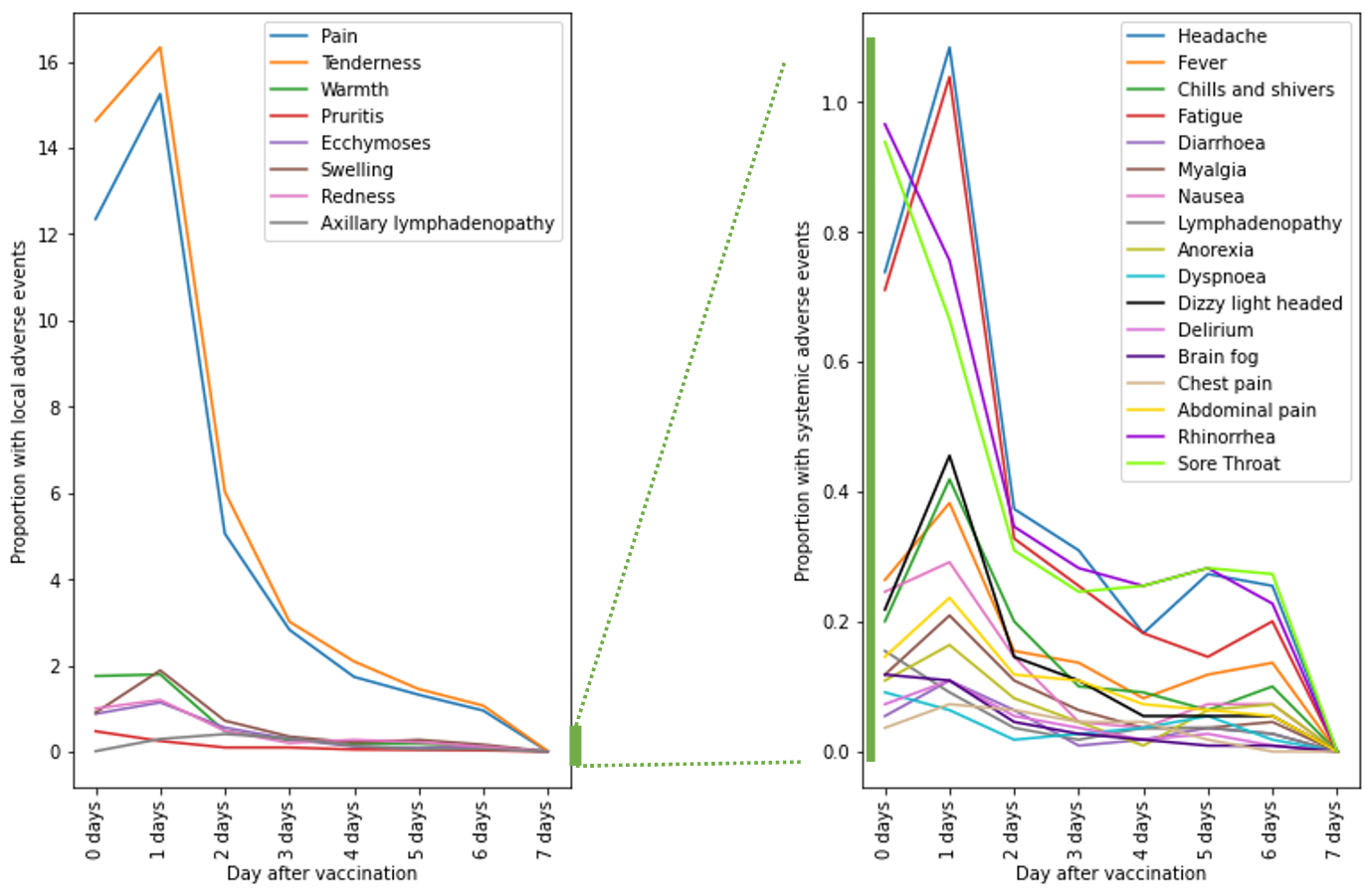
**
